# *Vital Signs:* Use of Recommended Health Care Measures to Prevent Selected Complications of Sickle Cell Anemia in Children and Adolescents — Selected U.S. States, 2019

**DOI:** 10.15585/mmwr.mm7139e1

**Published:** 2022-09-30

**Authors:** Laura A. Schieve, Gretchen M. Simmons, Amanda B. Payne, Karon Abe, Lewis L. Hsu, Mary Hulihan, Shammara Pope, Sarah Rhie, Brandi Dupervil, W. Craig Hooper

**Affiliations:** ^1^Division of Blood Disorders, National Center on Birth Defects and Developmental Disabilities, CDC; ^2^Division of Pediatric Hematology/Oncology, University of Illinois at Chicago, Chicago, Illinois.

## Abstract

**Introduction:**

Sickle cell disease (SCD), a group of inherited blood cell disorders that primarily affects Black or African American persons, is associated with severe complications and a >20-year reduction in life expectancy. In 2014, an expert panel convened by the National Heart, Lung, and Blood Institute issued recommendations to prevent or reduce complications in children and adolescents with the most severe SCD subtypes, known as sickle cell anemia (SCA); recommendations included 1) annual screening of children and adolescents aged 2–16 years with transcranial Doppler (TCD) ultrasound to identify those at risk for stroke and 2) offering hydroxyurea therapy to children and adolescents aged ≥9 months to reduce the risk for several life-threatening complications.

**Methods:**

Data from the IBM MarketScan Multi-State Medicaid Database were analyzed. TCD screening and hydroxyurea use were examined for 3,352 children and adolescents with SCA aged 2–16 years and continuously enrolled in Medicaid during 2019. Percentage change during 2014–2019 and variation by health subgroups were assessed. Analyses were stratified by age.

**Results:**

During 2014–2019, TCD screening increased 27% among children and adolescents aged 10–16 years; hydroxyurea use increased 27% among children aged 2–9 years and 23% among children and adolescents aged 10–16 years. However, in 2019, only 47% and 38% of children and adolescents aged 2–9 and 10–16 years, respectively, had received TCD screening and 38% and 53% of children and adolescents aged 2–9 years and 10–16 years, respectively, used hydroxyurea. For both prevention strategies, usage was highest among children and adolescents with high levels of health care utilization and evidence of previous complications indicative of severe disease.

**Conclusion and Implications for Public Health Practice:**

Despite increases since 2014, TCD screening and hydroxyurea use remain low among children and adolescents with SCA. Health care providers should implement quality care strategies within their clinics and partner with patients, families, and community-based organizations to address barriers to delivering and receiving recommended care.

## Introduction

Sickle cell disease (SCD), a group of inherited blood disorders characterized by abnormal hemoglobin, reduces life expectancy by >20 years (*1*). SCD primarily affects persons whose ancestors came from Africa, where malaria is endemic, because the carrier state (sickle cell trait, inheritance of a sickle cell gene from only one parent) confers a selective advantage by protecting against the harmful effects of malaria.* Thus, >90% of the estimated 100,000 persons in the United States with SCD are non-Hispanic Black or African American (Black), and an estimated 3%–9% are Hispanic or Latino (Hispanic) (*2*). In persons with SCD, red blood cells become rigid and deform into a crescent or sickle shape. Sickled cells die early and often become lodged in small blood vessels, compromising blood flow, which can lead to serious health problems. SCD-associated complications include anemia; acute and chronic pain; infections; pneumonia and acute chest syndrome^†^; stroke; and kidney, liver, and heart disease. Despite their extensive health care needs, many persons with SCD have difficulty accessing appropriate care and report feeling stigmatized and having their symptoms dismissed when they do seek care (*3*).

SCD comprises four main genotypes; among these, the hemoglobin SS and hemoglobin Sβ^0^-thalassemia genotypes are the more severe forms and are collectively referred to as sickle cell anemia (SCA). SCA accounts for an estimated 75% of SCD cases in the United States (*4*). In 2014, an expert panel convened by the National Institutes of Health’s National Heart, Lung, and Blood Institute (NHLBI) developed recommendations to prevent or reduce complications of SCD, several of which were specific to children and adolescents with SCA (*5*). Given that SCA is a common cause of childhood stroke (*6*), the panel recommended that children and adolescents aged 2–16 years with SCA be screened annually with transcranial Doppler (TCD) ultrasound to identify high cerebral blood velocity, an indicator of elevated stroke risk. Chronic blood transfusion therapy, the recommended intervention, substantially reduces stroke occurrence in children and adolescents identified as being at risk (*7*). The panel also recommended that children and adolescents aged ≥9 months with SCA (including asymptomatic children) be offered treatment with hydroxyurea, a medication shown to be efficacious in preventing or reducing severe pain episodes, acute chest syndrome, and other SCA-associated complications and increasing patient survival (*8*). Although the panel chose to recommend offering treatment as a means of opening discussion with families, it emphasized that an established evidence base supported the sustained benefits of hydroxyurea therapy for young persons with SCA without harmful effects on growth, development, female fertility, or increased risks for genetic mutations or cancer (*5*).

Previous studies documented underutilization of both TCD screening and hydroxyurea (*9*–*11*), and barriers to receipt of both interventions have been described (*12*–*15*). Barriers to TCD screening include limited radiology visit availability, distance between SCD clinics and radiology centers, providers’ lack of familiarity with TCD guidelines (including knowledge gaps among pediatric hematologists, neurologists, and primary care providers who care for children and adolescents with SCA), problems with care coordination (e.g., lack of timely information from radiology centers to providers), and provider concern that TCD screening will not affect outcomes because patients and families are often unable to sustain chronic blood transfusion therapy^§^ (*12*,*13*). Barriers to hydroxyurea use include patient and provider uncertainty regarding its effectiveness and fear of adverse effects (including perceived carcinogenesis potential), complexity of treatment regimen (which requires ongoing monitoring and laboratory visits), provider discomfort in managing hydroxyurea therapy, provider concern about lack of patient adherence, and high cost and lack of reimbursement (*13*–*15*). Recent studies on use of these prevention strategies are limited. This study examined TCD screening and hydroxyurea use among children and adolescents aged 2–16 years with SCA who were enrolled in Medicaid in 2019 and assessed changes since 2014.

## Methods

This study was conducted using the IBM MarketScan Treatment Pathways online analytic tool with data from the IBM MarketScan Multi-State Medicaid Database from January 1, 2010, to December 13, 2019, which includes medical claims data from approximately 24 million Medicaid enrollees from five to 15 states (the number of states varies by year). SCA was defined using an established algorithm, based on *International Classification of Diseases, Ninth Revision, Clinical Modification *and *International Classification of Diseases*, *Tenth Revision, Clinical Modification* diagnosis codes, previously validated to identify persons with SCA (*16*,*17*) (Supplementary Box, https://stacks.cdc.gov/view/cdc/120746). TCD screening and hydroxyurea use were defined based on procedure and pharmacy codes, respectively. A small proportion (5%) of children and adolescents receiving chronic blood transfusion therapy were excluded from analyses because transfusion therapy might be indicative of previous abnormal TCD results and hydroxyurea and chronic blood transfusion therapy might not be used concurrently. The final analytic sample included 3,352 children and adolescents with SCA who were continuously enrolled in Medicaid in 2019. To assess change over time, a sample of 3,858 children and adolescents continuously enrolled in Medicaid in 2014 were compared with the 2019 sample; the two samples had similar demographic and health profiles (Supplementary Table, https://stacks.cdc.gov/view/cdc/120747).

The proportions of TCD screening and hydroxyurea use and their corresponding 95% CIs were calculated for children and adolescents in 2014 and 2019; percentage change from 2014 to 2019 was also calculated. Differences between years were considered statistically significant if CIs did not overlap. Findings were stratified by age group (2–9 years and 10–16 years) during the respective study year.

In the 2019 sample, TCD screening and hydroxyurea use were examined within health care usage and disease severity subgroups; associations were assessed using prevalence ratios and 95% CIs. Two indicators of severe disease were examined, each defined by whether the child or adolescent had a severe complication (acute chest syndrome or multiple pain crises) in 2019 or any previous data year (2010–2018) (Supplementary Box, https://stacks.cdc.gov/view/cdc/120746). Data analyses were conducted using SAS software (version 9.4; SAS Institute). This activity was reviewed by CDC and was conducted consistent with applicable federal law and CDC policy.^¶^

## Results

From 2014 to 2019, TCD screening increased 27% among children and adolescents aged 10–16 years. Among children aged 2–9 years, TCD screening increased 9%, which was not statistically significant. Nonetheless, younger children had higher TCD screening rates than did older children and adolescents in both years; by 2019, proportions of children and adolescents who had received TCD screening were 47% and 38% among those aged 2–9 and 10–16 years, respectively (Figure).

**FIGURE:**
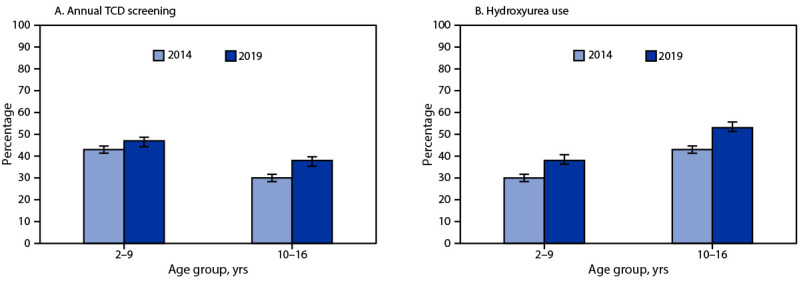
Percentage of annual transcranial Doppler ultrasound screening (A) and hydroxyurea use (B) among children and adolescents aged 2–16 years with sickle cell anemia* — selected U.S. states, 2014 and 2019 **Abbreviation**: TCD = transcranial Doppler. * With 95% CIs indicated by error bars.

In both age groups, TCD screening varied significantly by health indicators (Table 1). Among children aged 2–9 years, the highest TCD screening rates (>55%) were among children who had a recent hospitalization, 11–20 recent ambulatory care visits, a recent or previous hospitalization for acute chest syndrome, or two or more pain crises requiring hospitalization in the current year or a previous year. Among children and adolescents aged 10–16 years, the highest TCD screening prevalences (43%–48%) were observed among the same subgroups.

**TABLE 1:** Transcranial Doppler ultrasound screening among children and adolescents aged 2–16 years with sickle cell anemia continuously enrolled in Medicaid, within health indicator subgroups* — selected U.S. states, 2019

Health indicator	Children and adolescents who received TCD screening			
	Aged 2–9 yrs (n = 1,810)		Aged 10–16 yrs (n = 1,542)	
	No. (%)	PR (95% CI)	No. (%)	PR (95% CI)
**Total**	**845 (47)**	**NA**	**581 (38)**	**NA**
**Hospitalization during study year**				
Yes	349 (56)	1.3 (1.2–1.5)	266 (43)	1.3 (1.1–1.5)
No	496 (42)	Ref	315 (34)	Ref
**Ambulatory care visits during study year**				
0–10	449 (41)	Ref	271 (32)	Ref
11–20	275 (61)	1.5 (1.3–1.7)	195 (48)	1.5 (1.3–1.7)
21–30	60 (55)	1.3 (1.1–1.6)	51 (41)	1.3 (1.0–1.6)
>30	61 (41)	1.0 (0.8–1.2)	64 (39)	1.2 (1.0–1.5)
**Emergency department visits during study year**				
0	269 (46)	Ref	212 (36)	Ref
1–2	332 (46)	1.0 (0.9–1.1)	235 (39)	1.1 (0.9–1.2)
3–4	144 (46)	1.0 (0.9–1.2)	79 (38)	1.0 (0.8–1.3)
≥5	100 (54)	1.2 (1.0–1.4)	55 (39)	1.1 (0.9–1.4)
**Emergency department reliance**				
<20% of noninpatient visits	594 (51)	Ref	466 (41)	Ref
≥20% of noninpatient visits	251 (42)	0.8 (0.7–0.9)	115 (30)	0.7 (0.6–0.9)
**One or more hospitalizations for acute chest syndrome in any year (including current)**				
Yes	426 (57)	1.5 (1.3–1.6)	359 (45)	1.5 (1.3–1.8)
No	419 (39)	Ref	222 (30)	Ref
**Largest number of hospitalizations for pain crises in any year (including current)**				
0	236 (35)	Ref	99 (28)	Ref
1	292 (46)	1.3 (1.1–1.5)	156 (33)	1.2 (1.0–1.5)
2	170 (59)	1.7 (1.5–1.9)	135 (45)	1.6 (1.3–2.0)
>2	147 (67)	1.9 (1.7–2.2)	191 (47)	1.7 (1.4–2.1)

**Abbreviations:** NA = not applicable; PR = prevalence ratio; Ref = referent group; TCD = transcranial Doppler ultrasound.

* Children who had no ambulatory care visits and no emergency department visits during 2019 were not included in analyses of emergency department reliance.

From 2014 to 2019, hydroxyurea use increased significantly among children aged 2–9 years (27%) and children and adolescents aged 10–16 years (23%) (Figure). In 2019, hydroxyurea use was more prevalent among children and adolescents aged 10–16 years (53%) than among children aged 2–9 years (38%) and also varied significantly by health indicators (Table 2). Moreover, hydroxyurea use exceeded 60% among children and adolescents aged 10–16 years who had had a recent hospitalization, 11–30 recent ambulatory care visits, three or more recent emergency department visits, a recent or previous acute chest syndrome hospitalization, or two or more pain crises requiring hospitalization in the current year or a previous year. Among children aged 2–9 years, the prevalences of hydroxyurea use were highest (47%–58%) in the same subgroups, with one exception: there was little variation in hydroxyurea use by number of emergency department visits.

**TABLE 2:** Hydroxyurea use among children and adolescents aged 2–16 years with sickle cell anemia continuously enrolled in Medicaid, within health indicator subgroups* — selected U.S. states, 2019

Health indicator	Children and adolescents who received hydroxyurea			
	Aged 2–9 yrs (n = 1,810)		Aged 10–16 yrs (n = 1,542)	
	No. (%)	PR (95% CI)	No. (%)	PR (95% CI)
**Total**	**693 (38)**	**NA**	**821 (53)**	**NA**
**Hospitalization during study year**				
Yes	305 (49)	1.5 (1.3–1.7)	415 (68)	1.6 (1.4–1.7)
No	388 (33)	Ref	406 (44)	Ref
**Ambulatory care visits during study year**				
0–10	360 (33)	Ref	377 (45)	Ref
11–20	225 (50)	1.5 (1.3–1.7)	271 (66)	1.5 (1.3–1.6)
21–30	54 (49)	1.5 (1.2–1.8)	80 (65)	1.4 (1.2–1.7)
>30	54 (36)	1.1 (0.9–1.4)	93 (56)	1.3 (1.1–1.5)
**Emergency department visits during study year**				
0	215 (36)	Ref	278 (48)	Ref
1–2	268 (37)	1.0 (0.9–1.2)	316 (52)	1.1 (1.0–1.2)
3–4	131 (42)	1.1 (1.0–1.4)	130 (62)	1.3 (1.1–1.5)
≥5	79 (43)	1.2 (1.0–1.4)	97 (69)	1.5 (1.3–1.7)
**Emergency department reliance**				
<20% of noninpatient visits	484 (41)	Ref	622 (55)	Ref
≥20% of noninpatient visits	208 (34)	0.8 (0.7–0.9)	197 (51)	0.9 (0.8–1.0)
**One or more hospitalizations for acute chest syndrome in any year (including current)**				
Yes	348 (47)	1.5 (1.3–1.6)	514 (65)	1.6 (1.4–1.8)
No	345 (33)	Ref	307 (41)	Ref
**Largest number of hospitalizations for pain crises in any year (including current)**				
0	177 (26)	Ref	123 (34)	Ref
1	251 (40)	1.5 (1.3–1.8)	224 (47)	1.4 (1.1–1.6)
2	137 (48)	1.8 (1.5–2.1)	184 (62)	1.8 (1.5–2.1)
>2	128 (58)	2.2 (1.9–2.6)	290 (71)	2.1 (1.8–2.4)

**Abbreviations:** NA = not applicable; PR = prevalence ratio; Ref = referent group.

* Children who had no ambulatory care visits and no emergency department visits during 2019 were not included in analyses of emergency department reliance.

## Discussion

Although increases in both TCD screening and hydroxyurea use were observed during the 5 years after the NHLBI panel issued their recommendations (*5*), many children and adolescents with SCA were not receiving these potentially lifesaving interventions in 2019. Usage prevalences of both prevention strategies varied by age, with younger children less likely to use hydroxyurea and older children and adolescents less likely to have an annual TCD screen. Age differences were not explained by health characteristics: age prevalence patterns of both TCD screening and hydroxyurea use were consistent across all health care usage and disease severity subgroups examined. More specific reasons for the age differences cannot be examined with claims data. Usage of both prevention strategies was highest among children and adolescents with documentation of severe disease (i.e., those with manifest health care needs). Nonetheless, even among groups with the highest usage rates (younger children with an indication of severe disease for TCD screening and older children and adolescents with an indication of severe disease for hydroxyurea) a substantial proportion of children and adolescents for whom these interventions are indicated were not receiving them.

Previous studies document numerous barriers to receipt of both interventions (*12*–*15*). Promising quality-care initiatives to reduce some barriers have been reported. One SCD center leveraged electronic health records to enhance case management and improve TCD tracking and scheduling and enlisted support specialists to help young children remain relaxed during the procedure; they reported sustained increases in TCD screening, from 63% at baseline to >70% (*18*). A regional collaborative of SCD clinics reported a significant increase in hydroxyurea counseling (from 85% to 98%) after implementation of a program in which clinic staff members and families developed standardized approaches to track preventive care (*19*).

More than 90% of patients with SCD are Black, and 3%–9% are Hispanic (*2*); thus, racism and existing health care disparities compound barriers to care for children with SCA. Interpersonal racism, such as racist connotations, prejudice, discrimination, and bias toward patients with SCA, often results in inadequate care and prolonged suffering (*3*). Structural racism, policies that have led to unequal opportunities in housing, employment, health insurance, and research funding, keep disparities in place and contribute to adverse health outcomes. These challenges are exacerbated by poor access to health care for SCA, given the lack of providers with expertise or facilities with resources to treat SCA. Consequently, SCA patients might delay seeking care, and emergency department visits are common.

Preventing SCA-associated complications requires strategies to reduce racism and disparities. Health care providers can educate themselves, their colleagues, and their institutions about the unique and specific needs of persons with SCA, including how racism impedes optimal health care. They can advocate for and listen to their patients to better understand their needs. Population-based data are also critical to addressing gaps in health care. Data from the Sickle Cell Data Collection program, a state-based tracking system established by CDC in 2015 in California and Georgia, have directly informed health care decision-making. For example, a Georgia Sickle Cell Data Collection assessment that indicated that 10% of children and adolescents with SCD lived a >1-hour drive from any SCD specialty care option led to the opening of new mobile care clinics. Recently, the Sickle Cell Data Collection program expanded to 11 states, which collectively cover approximately 36% of persons with SCD in the United States.

The findings in this report are subject to at least five limitations. First, the sample for this analysis was limited to Medicaid enrollees from selected states; therefore findings are not generalizable to all U.S. children and adolescents with SCA. Nonetheless, previous assessments in two states indicate that most children and adolescents with SCD are covered by Medicaid.** Second, because the MarketScan Medicaid data files do not include information about which states participated each year, it was not possible to assess whether state variability partially explained changes in TCD screening and hydroxyurea use from 2014 to 2019. Third, socioeconomic data, such as income or parents’ level of education were also not available. Fourth, the SCA algorithm used in this study (*16*) maximized case-finding; thus, some children and adolescents with non-SCA genotypes might have been included in this analysis. However, the algorithm was most precise in classifying children and adolescents with SCA who had a previous severe complication, and the findings for those subgroups indicate that many children and adolescents with overt symptomatology are not receiving TCD screening or hydroxyurea. Finally, because hydroxyurea use was defined by the filling of a single prescription, the findings overestimate ongoing hydroxyurea use.

TCD screening is critical to stroke prevention in children and adolescents with SCA (*7*); hydroxyurea is efficacious in preventing serious complications (*8*), and numerous studies demonstrate the safety of its long-term use (*20*). The findings from this study highlight that health care for children and adolescents with SCA is fragmented. Health system accountability for evidence-based care can be built into electronic health records. Health care providers should implement quality care strategies to maximize TCD screening and hydroxyurea use and partner with patients, families, and community-based organizations to address barriers to care. Given that almost all SCA patients are Black or Hispanic (*2*), it is important that strategies include proactively addressing both interpersonal and structural racism. Finally, population-based surveillance data for SCA are currently limited to select states; expansion of surveillance coverage would allow CDC to better characterize disease outcomes and health care needs of those with SCA, and SCD overall, across the life span.

**Summary**

**What is already known about this topic?**

Sickle cell anemia (SCA), which primarily affects Black or African American persons, is associated with severe complications and reduced life expectancy. Among children and adolescents with SCA, transcranial Doppler (TCD) ultrasound screening identifies elevated risk for stroke, and hydroxyurea therapy can reduce the occurrence of several life-threatening complications.

**What is added by this report?**

During 2019, fewer than one half of Medicaid enrollees aged 2–16 years with SCA had a TCD screen. Fewer than one half of children aged 2–9 years used hydroxyurea and approximately one half of those aged 10–16 years used hydroxyurea.

**What are the implications for public health practice?**

Health care providers should implement quality care strategies and partner with patients, families, and community-based organizations to address barriers to care.

## Footnotes

* A more comprehensive summary of the genetics, epidemiology, and health outcomes associated with SCD, the health care needs of persons affected by SCD, and their challenges to receipt of optimal care can be found at National Academies of Sciences, Engineering, and Medicine. 2020. Addressing sickle cell disease: a strategic plan and blueprint for action. Washington, DC: The National Academies Press. https://doi.org/10.17226/25632

† Acute chest syndrome is a severe condition caused by sickling in the small pulmonary blood vessels and characterized by chest pain, cough, fever, hypoxia, and lung infiltrates.

§ Chronic blood transfusion therapy reduces the risk for stroke in children and adolescents with SCA identified to have elevated cerebral blood velocity through TCD screening. However, transfusion therapy is an intensive treatment and frequent transfusions have inherent risks including infection, iron overload, allergic reactions, alloimmunization, and hemolytic transfusion reactions. Therefore, children and adolescents who receive chronic transfusion therapy should be monitored closely and receive regular treatment (chelation therapy) to reduce excess iron buildup in the body.

¶ 45 C.F.R. part 46, 21 C.F.R. part 56; 42 U.S.C. Sect. 241(d); 5 U.S.C. Sect. 552a; 44 U.S.C. Sect. 3501 et seq.

**
https://www.cdc.gov/ncbddd/hemoglobinopathies/scdc-fact-sheet-medicaid-data.html
